# Impact of rotavirus vaccination on diarrheal hospitalizations in children younger than 5 years of age in a rural southern Mozambique

**DOI:** 10.1016/j.vaccine.2022.09.050

**Published:** 2022-10-19

**Authors:** Filomena Manjate, Llorenç Quintó, Percina Chirinda, Sozinho Acácio, Marcelino Garrine, Delfino Vubil, Tacilta Nhampossa, Eva D. João, Arsénio Nhacolo, Anelsio Cossa, Sérgio Massora, Gizela Bambo, Quique Bassat, Karen Kotloff, Myron Levine, Pedro L. Alonso, Jacqueline E. Tate, Umesh Parashar, Jason M. Mwenda, Inácio Mandomando

**Affiliations:** a Centro de Investigação em Saúde de Manhiça (CISM), Maputo 1929, Mozambique; b Global Health and Tropical Medicine (GHTM), Instituto de Higiene e Medicina Tropical (IHMT), Universidade Nova de Lisboa (UNL), 1349-008 Lisbon, Portugal; c Barcelona Institute for Global Health (ISGlobal), Hospital Clínic - Universitat de Barcelona, 08036 Barcelona, Spain; d Instituto Nacional de Saúde (INS), Ministério da Saúde, Marracuene 1120, Mozambique; e ICREA, Pg. Lluís Companys 23, 08010 Barcelona, Spain; f Pediatrics Department, Hospital Sant Joan de Déu, (University of Barcelona), 2, 08950, Barcelona, Spain; g Consorcio de Investigación Biomédica en Red de Epidemiología y Salud Pública (CIBERESP), Madrid, Spain; h Center for Vaccine Development (CVD), University of Maryland School of Medicine, Baltimore, MD 21201, USA; i Global Malaria Programme, World Health Organization, 1211 Geneva, Switzerland; j Centers for Disease Control and Prevention (CDC), Atlanta, GA 30333, USA; k African Rotavirus Surveillance Network, Immunization, Vaccines and Development Program, World Health Organization, Regional Office for Africa, Brazzaville P.O. Box 2465, Congo

**Keywords:** Mozambique, Rotavirus, Diarrhea, Manhiça, Vaccine impact

## Abstract

**Background:**

Rotavirus vaccine (Rotarix®) was introduced in Mozambique through its Expanded Program of Immunization in September 2015. We assessed the impact of rotavirus vaccination on childhood gastroenteritis-associated hospitalizations post-vaccine introduction in a high HIV prevalence rural setting of southern Mozambique.

**Methods:**

We reviewed and compared the trend of hospitalizations (prevalence) and incidence rates of acute gastroenteritis (AGE), and rotavirus associated-diarrhea (laboratory confirmed rotavirus) in pre- (January 2008–August 2015) and post-rotavirus vaccine introduction periods (September 2015–December 2020), among children <5 years of age admitted to Manhiça District Hospital.

**Results:**

From January 2008 to December 2020, rotavirus vaccination was found to contribute to the decline of the prevalence of AGE from 19% (95% CI: 18.14–20.44) prior to the vaccine introduction to 10% (95% CI: 8.89–11.48) in the post-introduction period, preventing 40% (95 % IE: 38–42) and 84% (95 % IE: 80–87) of the expected AGE and laboratory confirmed rotavirus cases, respectively, among infants. Similarly, the overall incidence of rotavirus was 11.8-fold lower in the post-vaccine introduction period (0.4/1000 child-years-at-risk [CYAR]; 95% CI: 0.3–0.6) compared with the pre-vaccination period (4.7/1000 CYAR; 95% CI: 4.2–5.1) with the highest reduction being observed among infants (16.8-fold lower from the 15.1/1000 CYAR in the pre-vaccine to 0.9/1000 CYAR in the post-vaccine eras).

**Conclusions:**

We documented a significant reduction in all-cause diarrhea hospitalizations and rotavirus positivity after vaccine introduction demonstrating the beneficial impact of rotavirus vaccination in a highly vulnerable population.

## Highlights

• Rotavirus vaccine introduction in Mozambique reduced significantly acute gastroenteritis hospitalizations and rotavirus-associated hospitalizations.
• Our data demonstrated similar reduction in rotavirus detection rates such as that observed in urban settings of Mozambique (Maputo, Beira, Quelimane and Nampula cities), demonstrating the importance of the surveillance systems in rural setting like Manhiça.
• In our study we reported a decline of rotavirus-associated AGE in the age groups of children not covered by the vaccination program (older than 2 years of age), which supports the beneficial effect of vaccination.

## 1 Introduction

Despite the worldwide deployment of rotavirus vaccines into the expanded program of immunizations (EPI), rotavirus remains the leading cause of severe gastroenteritis among children <5 years of age. Estimates from 2016 showed that rotavirus was responsible for 128,500 deaths (95% uncertainty interval [UI], 104 500–155 600) of children younger than 5 years worldwide, approximately 82% (104 733; 95% UI: 83 406–128 842) of these deaths occurred in sub-Saharan Africa [1]. The Global Enteric Multicenter Study (GEMS), which aimed to quantify the burden and etiology of moderate-to-severe diarrhea (MSD) in infants and young children living in sub-Saharan Africa (including Mozambique) and south Asia, confirmed rotavirus as the leading pathogen associated with MSD, and with the highest attributable fraction observed among Mozambican children [2].

In the rural district of Manhiça, southern Mozambique, data from GEMS showed that rotavirus was responsible for an attributable fraction of approximately 35% of all diarrheal cases requiring admission [2] and 20% of ambulatory diarrheal cases [3] in infants, suggesting that effective vaccine would contribute to prevent these cases. These data were important for supporting the Mozambican Ministry of Health’s application to the Gavi, the vaccine alliance, for the introduction of the rotavirus vaccine (Rotarix®; GlaxoSmithKline Biologicals, Rixensart, Belgium), into the National EPI, subsequently launched at countrywide level in September 2015 [4]. Rotarix, a monovalent vaccine, composed by human G1[P8] strain is administered orally in two doses, at 2 and 3 months of age in Mozambican EPI scheme [5]; and the vaccine coverage was 73% in 2021, 6% less than the coverage rate of 2020 [6]. Data on vaccine coverage from the study area showed higher number of children immunized, compared to the expected (12342 vaccinated of the 10,176 expected in 2020; while in 2021 there were vaccinated 11,635 children of the 7248 expected) suggestion over 100% coverage which may be because of lack of precise denominator (unpublished data from the district health services of Manhiça-EPI annual report). After rotavirus introduction in Mozambique, the *Centro de Investigação em Saúde de Manhiça* (CISM) in Manhiça district have been monitoring the impact of rotavirus vaccine [4].

Countries that have introduced rotavirus vaccine in their EPI have shown a significant beneficial effect of the vaccine on the reduction of both diarrheal hospitalization cases and rotavirus-associated diarrhea [7], [8]. Mozambique is not an exception where early impact of rotavirus vaccination was demonstrated showing significantly decline of rotavirus-associated diarrhea from 40.2% in 2014 (pre-vaccine) to 13.5% in 2017 (post-vaccine) in urban settings of three provinces (Maputo, Beira and Nampula) [4], where the population has access to many healthcare services, live in improved households, have access to piped water and improved sanitation, do not practice agriculture as a source of living [4]. In addition, rotavirus vaccine was found to be cost-effective in Mozambique, preventing 4,628 deaths, and averting US$3.1 million in healthcare costs from 2016 to 2020 [9].

Although the impact of rotavirus was demonstrated in urban areas of Mozambique, it is possible that the impact may differ from that potentially achievable in a rural area such as Manhiça where HIV prevalence is amongst the highest in the world, where 39.7% of prevalence was documented among adults in 2012 [10], while 30% of children HIV positive were followed at HDM [11], and 25% of hospitalized children with MSD were HIV positive [12]. Furthermore, differences in socio-demographic characteristics of the population may affect the vaccine impact [10]. Thus, we aimed to evaluate the contribution of rotavirus vaccine in the reduction of diarrheal hospitalizations and rotavirus positivity among children younger than 5 years of age in a rural area of Manhiça after vaccine introduction.

## 2 Methodology

### 2.1 Study area and population

This study was conducted by CISM in the rural district of Manhiça, located 80 Km north of Maputo, in southern Mozambique [13]. Briefly, the district covers an area of ∼2380 km^2^ and has a subtropical climate, with two distinct seasons: warm and rainy from November to April; and a cool and dry during the rest of the year. In 1996, CISM implemented in the district an active and continuous Health and Demographic Surveillance System (HDSS), which has regular updates of demographic events for the entire surveyed population, and currently covering approximately 201, 845 inhabitants in 46, 726 households [14]. The CISM’s HDSS is linked to a morbidity surveillance system (MSS) ongoing since 1998 at the Manhiça District Hospital (MDH), a 150-bed capacity (and a 34-bed specific pediatric ward) referral hospital in the district of Manhiça and in other five peripheral health facilities within the district [13], [15].

The MSS documents all outpatients and inpatients visits of children under 15 years of age. Standardized forms are routinely completed during the visits, and include demographic, clinical and laboratory data. In addition to the morbidity system, data on diarrheal disease agents in Manhiça was provided through the GEMS study until 2012, GEMS was a case-control study of MSD and less-severe diarrhea (LSD only one year) in all admitted children <5 years of age, conducted between December 2007 and November 2012. Details of the GEMS have been previously described [2], [3], [16]. After the introduction of the vaccine (September 2015), a laboratory-based surveillance of diarrheal diseases was established to assess the etiologies of diarrhea including rotavirus using the same protocol as GEMS. The surveillance also aimed to monitor rotavirus vaccine impact.

### 2.2 Patient enrollment and sample collection

Diarrheal cases were passively detected through the MSS, where children younger than 5 years of age admitted at the MDH throughout the study period (January 2008–December 2020) were captured. For the analysis of impact of the vaccine, the post-introduction period was defined from June 2016 to December 2020, while pre-vaccine period was considered from January 2008 to November 2012. The period of December 2012 and September 2015 was not considered for the analysis, as there was no laboratory testing for pathogens detection. Rotavirus testing was done for children enrolled in the GEMS and diarrheal surveillance platform, implemented within the context of the surveillance of rotavirus and other enteropathogens in children <5 years of age in Manhiça. In both studies, children were enrolled if fulfilling at least one of the following criteria for MSD: Sunken eyes; loss of skin turgor (abdominal skin pinch with slow [≤2 s] or very slow [>2 s] recoil); intravenous hydration administered or prescribed; dysentery (visible blood in loose stools); or admission to the hospital with diarrhea or dysentery. To be included in the incidence analysis, the episode had to be new (onset after ≥15 diarrhea-free days) and acute (onset within the previous 7 days) and from a child identified in the census, with confirmation that he/she was actually living in the study area on the day of the episode. Written informed consent was sought from the child's representative before compilation of clinical, anthropometric measurements, epidemiological information and sample collection.

### 2.3 Laboratory testing

Laboratory based investigation of rotavirus was conducted between January 2008 and November 2012 during the GEMS study and from September 2015 to December 2020 as part of routine diarrheal surveillance. The laboratory procedures for the extensive microbiological investigations, conducted to characterize each diarrheal episode have been previously described [2], [3], [16]. Rotavirus was detected using commercial immunoassays kit (ProSpectTM Rotavirus, Oxoid, UK) as described by the manufacturer. Due to resources’ constraints, there was no active laboratory-based rotavirus investigation between November 2012 and August 2015.

### 2.4 Data management and statistical analysis

Data from the MSS were double entered in the Visual FoxPro or OpenClinica data management software’s and checked for their consistency. Laboratory data were entered in a Laboratory Information System (ServoLab, Germany) and a study master database was created to combin**e** clinical/epidemiological and laboratory data. All-cause admissions, acute gastroenteritis (AGE) and malnutrition (MNUT) admissions were obtained from the ongoing morbidity surveillance system [13]. Having an International Classification of Disease, Version 10 (ICD-10) codes for acute gastroenteritis listed among the diagnoses that can be described as being responsible for the patient's hospitalization was used for outcome ascertainment of AGE as described (Table S1) [17].

The exposure time intervals of each individual in the study area were obtained from the HDSS running in the study area since 1996 [18]. To estimate the incidences, the time at risk was calculated as the number of person years at risk since the beginning of the time at risk until the end of follow-up. The beginning of time at risk was defined for each child as the first day of study period (January 1, 2008) or date of birth, whatever occurred later. The end of follow-up was defined for each child as the last day of study period (December 31, 2020), the day he/she turned 5 or the date of death, whatever occurred first. An arbitrary lag of 15 days was applied after each episode. Children did not contribute to the time at risk or to the cases during the lag periods. Episodes were identified by passive case detection, which underestimates the true number of episodes and therefore incidences are in fact “minimum incidences”. Months are defined as 30.4 days, incidences are expressed as episodes per 1000 CYAR where CYAR is “Children-Years At Risk” (i.e. episodes per 365.25 days) in the tables and per 1000 CMAR (Children-Months At Risk) in the figures.

Logistic regression models were estimated to compare the prevalence of all cause admission, AGE, and laboratory confirmed rotavirus (LC-RV) among all children under five years admitted at the MDH, whether or not from the study area. For children from the study area, negative binomial regression models were estimated to compare incidence rates. These models were estimated with random intercept to take into account repeated measures, since children could change age or exposure during follow-up.

The vaccine impact was estimated assessing the longitudinal effect of rotavirus vaccination through the trend of monthly incidence rates of hospitalizations, AGE, malnutrition and LC-RV among children younger than 5 years of age. An interrupted time-series analysis [19], was performed with monthly data on cases of AGE, malnutrition and LC-RV to measure the impact of the intervention assuming a negative binomial distribution of the number of monthly counts. Models for incidence rates (only for children from the study area) were estimated using the amount of time each child was living in the study area according to the DSS as the amount of exposure over which the events were observed for each month and were estimated with time-at-risk included as an offset.

The regression models included terms for the intervention, secular trends for the periods before/after vaccine implementation and a regular cyclical component for seasonality [20], [21]. Models were estimated with robust standard errors adjusted for clustering on whether child came from the study area in models for absolute counts and clustering on study region in models for incidence rates.

Counterfactual estimates and their variance based on pre-vaccine parameters from the regression models were used to calculate the number of cases expected in the post-vaccine period if the intervention had not been implemented. Their marginal effects, estimated holding the amount of exposure (admissions or time-at-risk) at the inter-cluster monthly means, were used for the figures. Also, the linear trend was estimated as the marginal effect of the regression model estimates, holding the amount of exposure and seasonal covariate at the means of the pre- and post-vaccine periods.

At each month after the intervention roll-in period, 1000 predicted case counts were calculated based on 1000 random draws from the predictive distribution of the model parameters. The median number of cases derived from those simulations represented the number of cases expected in the post-vaccine introduction period. The 2.5th and 97.5th percentiles of those simulations represented the upper and lower 95 percent interval estimates (95 % IE) around the point estimates. The vaccine impact was calculated as the relative difference between expected and actual case counts that are (expected-observed)/expected [21]. An intervention roll-in period of 9 months was considered for analysis. This roll-in period was excluded for the estimation of the regression models, as well as the no rotavirus-surveillance period for the estimation of the specific models for rotavirus [22]. All analyses, data manipulation, and implementations were done using Stata software [23].

### 2.5 Ethical approval

The GEMS study protocol was approved by The National Bioethics Committee for Health (CNBS) of Mozambique (reference 11/CNBS/07; IRB 00002657), 19 February 2007. The protocol of surveillance of rotavirus and other enteropathogens in children <5 years of age was also approved by the National Bioethics Committee for Health (CNBS) of Mozambique also approved the (reference 209/CNBS/15; IRB00002657), 22 July 2015.

## 3 Results

### 3.1 Burden of acute gastroenteritis (AGE)

In the study population, the overall mean prevalence of all-cause AGE was 13.7% (95% CI: 13.1–14.2) with a significant decline from 15.0% (95% CI: 14.4–15.8) before the vaccine introduction to 10.1% (95% CI: 9.2–11.0)**,** five years after the vaccine was introduced. Such decline was particularly prominent among infants, where the prevalence dropped from 19.2% (95% CI: 18.1–20.4) to 10.1% (95% CI: 8.9–11.4, *p* < 0.0001) as shown in Table 1. A similar trend was observed on the incidence of AGE dropping from 30.2 cases per 1000 children-years-at-risk (95% CI: 27.7–33.0) in pre-vaccine period to 5.9 cases per 1000 children-years-at-risk (95% CI: 4.9–6.8), yielding an incidence rate ratio (IRR) of 0.2 (95% CI: 0.2–0.2; *p < 0.0001*) after vaccine introduction period among infants. A significantly reduction was also observed among children aged 12–23 months with an IRR = 0.1 95% CI: (0.1–0.2; *p < 0.0001*, Table 2).

**Table 1:** Estimated prevalence of all-cause of acute gastroenteritis among children younger than 5 years of age stratified by age groups, admitted to the Manhiça District Hospital, Manhiça Mozambique, January 2008 – December 2020.

Age category/Exposure	Admissions from all causes	Episodes of AGE	Rate estimates		Model estimates		p-value
			Prevalence (%)	95% Conf. Interval	Odds Ratio	95% Conf. Interval	
**0**–**11 months**							
Pre-vaccine introduction	4614	889	19.2	(18.1, 20.4)	1		
Post-vaccine introduction	2152	218	10.1	(8.9, 11.4)	0.4	(0.4,0.6)	<0.0001
Total	6766	1107	16.3	(15.4, 17.2)	–		
**12**–**23 months**							
Pre-vaccine introduction	2998	579	19.3	(17.9, 20.8)	1		
Post-vaccine introduction	928	138	14.9	(12.6, 17.3)	0.7	(0.6,0.9)	0.0023
Total	3926	717	18.2	(17.0, 19.5)	–		
**24**–**59 months**							
Pre-vaccine introduction	3464	202	5.8	(5.0, 6.7)	1		
Post-vaccine introduction	1205	79	6.6	(5.2, 8.1)	1.1	(0.8,1.4)	0.3626
Total	4669	281	6.0	(5.3, 6.7)	–		
**All age strata**							
Pre-vaccine introduction	11,076	1670	15.0	(14.4, 15.8)	1		
Post-vaccine introduction	4285	435	10.1	(9.2, 11.0)	0.6	(0.5,0.7)	<0.0001
Total	15,361	2105	13.7	(13.1, 14.2)	–		

AGE: Acute gastroenteritis.

**Table 2:** Estimated incidence rates of all-cause acute gastroenteritis among children younger than 5 years of age stratified by age groups, admitted to the Manhiça District Hospital, Manhiça Mozambique January 2008 – December 2020.

Age category/Exposure	Subjects	Episodes of AGE	Time At Risk (CYAR)	Rate estimates		Model estimates		p-value
				Incidence Rate (per 1000 CYAR)	95% Conf. Interval	Incidence Rate Ratio	95% Conf. Interval	
**0**–**11 months**								
								
Pre-vaccine introduction	21,259	485	16022.42	30.2	(27.7,33.0)	1		
Post-vaccine introduction	32,682	147	25142.01	5.9	(4.9,6.9)	0.2	(0.2, 0.2)	<0.0001
Total	53,941	632	41164.43	15.3	(14.2, 16.6)	–		
								
**12**–**23 months**								
Pre-vaccine introduction	20,584	361	15628.27	23.1	(20.9,25.6)	1		
Post-vaccine introduction	33,187	93	25384.92	3.7	(2.9,4.5)	0.2	(0.1, 0.2)	<0.0001
Total	53,771	454	41013.19	11.0	(10.1,12.1)	–		
								
**24**–**59 months**								
Pre-vaccine introduction	27,732	137	46080.63	2.9	(2.5,3.5)	1		
Post-vaccine introduction	49,386	63	79833.25	0.7	(0.6,1.0)	0.3	(0.2, 0.4)	<0.0001
Total	77,118	200	125913.88	1.5	(1.3,1.8)	–		
								
**All age strata**								
Pre-vaccine introduction	37,350	983	77731.32	12.7	(11.9,13.5)	1		
Post-vaccine introduction	63,372	303	130360.19	2.3	(2.0,2.6)	0.1	(0.2, 0.2)	<0.0001
Total	96,351	1286	208091.5	6.1	(5.9,6.5)	–		

AGE: Acute gastroenteritis.

### 3.2 Laboratory confirmed rotavirus cases

The overall prevalence of LC-RV showed a significant drop in the first two age groups. A larger decline was seen among infants from a baseline of 27.9% (95% CI: 25.0–31.0) to 9.6% (95% CI: 6.1–14.4) in the post-vaccine introduction period (Table 3). Additionally, among infants there was a drop in the LC-RV incidence rate yielding an IRR of 0.1 (95% CI: 0.1–0.1; *p* < 0.0001) declining from the baseline estimates of 15.1 cases per1000 CYAR (95% CI: 13.3–17.1) to 0.9 cases per 1000 CYAR (95% CI: 0.6–1.7) (Table 4).

**Table 3:** Estimated prevalence of laboratory confirmed rotavirus among children younger than 5 years stratified by age groups admitted to the Manhiça District Hospital, Manhiça Mozambique January 2008 – December 2020.

Exposure	Episodes of AGE	Laboratory confirmed rotavirus	Rate estimates		Model estimates		p-value
			Prevalence (%)	95% Conf. Interval	Odds Ratio	95% Conf. Interval	
**0**–**11 months**							
Pre-vaccine introduction	889	248	27.9	(25.0, 31.0)	1		
Post-vaccine introduction	218	21	9.6	(6.1,14.4)	0.3	(0.2,0.4)	<0.0001
Total	1107	269	24.3	(21.8, 26.9)	–		
**12**–**23 months**							
Pre-vaccine introduction	579	92	15.9	(13.0, 19.1)	1		
Post-vaccine introduction	138	12	8.7	(4.6, 14.7)	0.5	(0.3,0.9)	0,0339
Total	3926	104	2.7	(2.1,3.2)	–		
**24**–**59 months**							
Pre-vaccine introduction	202	43	21.3	(15.9,27.6)	1		
Post-vaccine introduction	79	17	21.5	(13.0,32.2)	1.0	(0.5,1.9)	0.9660
Total	4669	60	1.2	(0.9,1.6)	–		
**All age strata**							
Pre-vaccine introduction	1670	383	22.9	(20.9, 25.0)	1		
Post-vaccine introduction	435	50	11.5	(8.7,14.9)	0.4	(0.3,0.6)	<0.0001
Total	2105	433	20.6	(18.9, 22.4)	–

**Table 4:** Estimated incidence rates of laboratory confirmed rotavirus among children younger than 5 years of age, stratified by age group, admitted to the Manhiça District Hospital, Manhiça Mozambique January 2008 – December 2020.

Exposure	Subjects	Episodes	Time At Risk (CYAR)	Rate estimates		Model estimates		p-value
				zy	95% Conf. Interval	Incidence Rate Ratio	95% Conf. Interval	
**0**–**11 months**								
Pre-vaccine introduction	21,021	240	15864.92	15.1	(13.3,17.1)	1		
Post-vaccine introduction	18,661	14	14321.27	0.9	(0.6,1.7)	0.1	(0.1,0.1)	<0.0001
TOTAL	39,682	254	30186.19	8.4	(7.4,9.6)	–		
**12**–**23 months**								
Pre-vaccine introduction	20,332	87	15454.92	5.6	(4.6,7.0)	1		
Post-vaccine introduction	18,780	9	14384.88	0.6	(0.3,1.2)	0.1	(0.1,0.2)	<0.0001
TOTAL	39,112	96	29839.81	3.2	(2.6,3.9)	–		
**24**–**59 months**								
Pre-vaccine introduction	27,339	32	45456.93	0.7	(0.5,1.0)	1		
Post-vaccine introduction	27,589	4	44617.99	0.1	(0.03,0.2)	0.1	(0.1,0.4)	0.0001
TOTAL	54,928	36	90074.92	0.4	(0.2,0.5)	–		
All age strata								
Pre-vaccine introduction	36,840	359	76776.78	4.7	(4.2,5.1)	1		
Post-vaccine introduction	35,570	27	73324.14	0.3	(0.2,0.5)	0.1	(0.1,0.1)	<0.0001
TOTAL	68,164	386	150100.93	2.6	(2.3,2.8)	–		

### 3.3 Burden of malnutrition

The overall mean prevalence of malnutrition in the population was 14.0% (95% CI: 13.4–14.6) with a significant decline from 14.8% (95% CI: 14.1–15.5) before vaccine introduction to 11.9% (95% CI: 11.0–13.0, *p < 0.0001*), five years after the vaccine introduction. The decline was remarkably significant in children 24–59 months, with 8.9% (95% CI: 7.8–9.9) in before vaccine introduction to 5.9% (95% CI: 4.6–7.3, *p < 0.0001*, Table S1) after vaccine introduction. A significant decline of malnutrition incidence was observed among all ages per 1000 children-years-at-risk before and after vaccine introduction periods, with a higher reduction observed in children from 24 to 59 months of age, with an IRR = 0.1, 95% CI (0.1–0.2, *p* < 0.0001, Table S2).

### 3.4 Impact of rotavirus vaccine on the prevalence and incidence rates of hospital admissions, AGE and rotavirus-associated diarrhea adjusted for seasonality

Overall, before the vaccine introduction, analysis of longitudinal effects of the vaccine demonstrated that the prevalence of all-cause admissions and AGE appeared to decrease significantly every month (with negative coefficients in the regression equation), while LC-RV was increasing (p-value < 0.0001). Additionally, after vaccine introduction, there was an immediate effect of decrease in all-cause admissions and LC-RV (both with p-value < 0.0001), followed by a significant decrease in the monthly trend of admissions due to AGE and LC-RV relative to the pre-intervention trend (p-value = 0.0351 and p-value <0.0001 respectively, Table 5). A significant monthly decrease was seen in the number of LC-RV hospitalizations in all age strata with *p* = 0.0003 in children <12 months, *p* < 0.0001 from 12 to 23 and 23–24 months of age (Tables S3A-C). Additionally, exploratory analysis of rotavirus detection by months shows a slight tendency of variations of monthly positivity, with high rates observed between June-August before vaccine and between September-October after vaccine introduction (Fig. S5).

**Table 5:** Longitudinal effect of rotavirus vaccination on the number of hospitalizations, acute gastroenteritis and laboratory confirmed rotavirus adjusted for seasonality among children < 5 years of age admitted to the Manhiça District Hospital, Manhiça Mozambique January 2008 – December 2020.

Variable	Prevalence			Incidence		
	Coef.	(95% Conf. Interval)	p-value	Coef.	(95% Conf. Interval)	p-value
**All cause admissions**						
Baseline level (Intercept)	4.70	(4.26, 5.14)	< 0.0001	−4.66	(-4.79, −4.53)	< 0.0001
Baseline monthly trend	−0.01	(-0.01, 0)	0.0200	−0.01	(-0.01, −0.01)	< 0.0001
Level change after RV-vaccine introduction	−0.37	(-0.39, −0.35)	< 0.0001	−0.35	(-0.48, −0.23)	< 0.0001
Monthly trend change after RV-vaccine introduction	0	(-0.02, 0.01)	0.5705	0.01	(0.0, 0.01)	0.0180
**Diagnosis of acute gastroenteritis**						
Baseline level (Intercept)	2.96	(2.41, 3.51)	< 0.0001	−6.39	(-6.52, −6.27)	< 0.0001
Baseline monthly trend	−0.01	(-0.02, 0)	0.0036	−0.02	(-0.02, −0.01)	< 0.0001
Level change after RV-vaccine introduction	−0.30	(-0.93, 0.33)	0.3480	0.02	(-0.26, 0.30)	0.8820
Monthly trend change after RV-vaccine introduction	−0.01	(-0.02, 0)	0.0351	0	(-0.01, 0.01)	0.8354
**Laboratory confirmed rotavirus**						
Baseline level (Intercept)	1.11	(-0.61, 2.83)	0.2049	−8.26	(-8.59, −7.94)	< 0.0001
Baseline monthly trend	0.01	(0.01, 0.01)	<0.0001	0.01	(0, 0.02)	0.0322
Level change after RV-vaccine introduction	−1.92	(-2.33, −1.50)	<0.0001	−3.07	(-3.92, −2.23)	< 0.0001
Monthly trend change after RV-vaccine introduction	−0.02	(-0.03, −0.02)	<0.0001	−0.04	(-0.06, −0.01)	0.0047

Nonetheless, in children younger than 5 years of age, the incidence rate of all-cause admission and AGE significantly decrease every month (with negative coefficients in the regression equation), while LC-RV was increasing (p-value < 0.0001) before vaccine introduction. In addition, after vaccine introduction, there was observed an immediate effect of decrease in all-cause admissions and LC-RV (both with p-value < 0.0001). Followed by a significant decrease in the monthly trend of LC-RV relative to the pre-intervention trend (p-value = 0.0047). (Table 5). In addition to the incidence rates in all age strata age, children <12 months, 12–23 months and 24–59 months showed a base line change of LC-RV after vaccine introduction (*p < 0.0001*, *p < 0.0001*, *p = 0.0002* respectively) (Tables S3A-C).

### 3.5 Trends over time of prevalence and incidence rates of hospital admissions, AGE, malnutrition and rotavirus-associated diarrhea

**Fig. 1:**
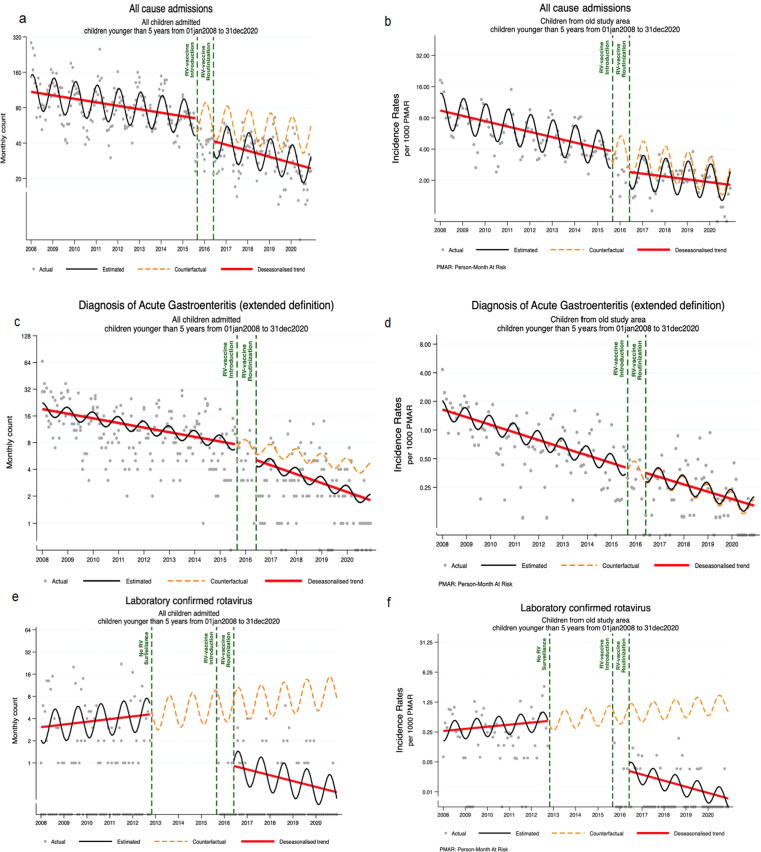
Trends of prevalence and incidence rates among children<5 years of age from Manhiça District, Manhiça Mozambique January 01, 2008 – December 2020. (a) Trends over time of all hospital admissions (b) trend over time of the incidence rate of all hospital admission due to acute gastroenteritis (c) trend over time of hospital admissions due to acute gastroenteritis (d) trends over time of the incidence rate of hospital admission due to acute gastroenteritis (e) Trends over time of rotavirus confirmed cases (f) trend over time of incidence rates of laboratory confirmed rotavirus.

The overall analysis of trends over time prevalence and incidence rates of hospitalizations, AGE, LC-RV and malnutrition are shown in Fig. 1A-f and Fig. S4A-B. There was a consistent and significant reduction of the trend over time prevalence of hospitalizations, AGE, malnutrition and LC-RV throughout the study period (Fig. 1A, C, E and Fig S4A), with possible evidence of the role of the vaccine in the reduction of hospitalization, AGE and LC-RV (Fig. 1 A, C, E). Even though, the vaccine was estimated to prevent 41% (95 % IE: 40–43, Table 6) of the 593 expected admissions with AGE among children <5 years of age and the evidence of possible effect of vaccination was observed in the LC-RV, preventing approximately 94% (95 % IE: 93–95) of expected rotavirus confirmed cases (Table 6).

**Table 6:** Number of cases expected and averted by rotavirus vaccine and the impact of the vaccination on the number of acute gastroenteritis (AGE), malnutrition and laboratory confirmed cases in children < 5 years of age and stratified by age groups (0–11, 12–23 and 24–59 months), Manhiça District, Manhiça Mozambique January 2008 – December 2020.

	Expected episodes n (95 % IE)	Observed episodes	Averted episodes n (95 % IE)	Relative difference % (95 % IE)
**Overall**				
AGE	593 (577, 609)	349	244 (228, 260)	41 (40, 43)
Incidence rate of AGE	209 (209, 2010)	218	−9 (−9,−8)	−4 (−4,−4)
Incidence rate of malnutrition	134 (134, 135)	217	−83 (−83,−82)	−61 (−62,−61)
Laboratory confirmed rotavirus	833 (687, 984)	50	783 (637, 934)	94 (93, 95)
Incidence rate of laboratory confirmed rotavirus	1508 (1492, 1523)	27	1481 (1465, 1496)	98 (98, 98)
**0**–**11 months**				
AGE	289 (279, 299)	172	117 (107, 127)	40 (38, 42)
Incidence rate of AGE	110 (109,111)	101	9 (8, 10)	8 (8, 9)
Incidence rate of malnutrition	36 (35, 36)	71	−35 (−36,−35)	−99 (−101, −98)
Laboratory confirmed rotavirus	181 (145, 217)	29	152 (116,188)	84 (80, 87)
Incidence rate of laboratory confirmed rotavirus	407 (401, 412)	14	393 (387, 398)	97 (97, 97)
**12**–**23 months**				
AGE	184 (182, 185)	114	70 (68, 67)	38 (37, 38)
Incidence rate of AGE	80 (80,81)	70	10 (10, 11)	13 (12, 13)
Incidence rate of malnutrition	97 (97, 98)	119	−22 (−22,−21)	−22 (−23,−22)
Laboratory confirmed rotavirus	791 (566, 1013	20	771 (546, 993)	97 (96, 98)
Incidence rate of laboratory confirmed rotavirus	1820 (1783, 1856)	9	1811 (1774, 1847)	100 (99, 100)
**24**–**59 months**				
AGE	81 (75, 88)	63	18 (12, 25)	22 (16, 28)
Incidence rate of AGE	21 (20, 21)	47	−26 (−27, −26)	−129 (−131, −127)
Incidence rate of malnutrition	13 (12, 13)	27	−14 (−15,−14)	−116 (−118, −114)
Laboratory confirmed rotavirus	815 (342, 1290)	17	798 (325, 1273)	98 (95, 99)
Incidence rate of laboratory confirmed rotavirus	4906 (4668, 5109)	4	4902 (4664, 5105)	100 (100, 100)

In contrast, the incidence rates of AGE were steadily similar before and after vaccine introduction, whereas the incidence rates of LC-RV had a significant shift after the vaccine introduction (Fig. 1D, F). Age stratified analysis on the trend over time proportions and incidence rates of hospitalization and the related incidence rate for AGE, malnutrition and LC-RV cases are shown in Figs. S1A-F to S4A-H, these longitudinal effects and estimated vaccine impact analysis showed more than 50% of LC-RV cases were prevented by the vaccine, including 98% (95 % IE: 95–99) of prevented cases among children aged 24–59 months (Table 6).

## 4 Discussion

Following the introduction of the monovalent rotavirus vaccine, we witnessed the acceleration of decline of all-cause admissions, diarrheal disease and more specifically rotavirus-associated AGE hospitalizations at a large referral hospital in Manhiça District, Southern Mozambique, over five consecutive years after vaccine introduction (September 2015 – December 2020). Although declining AGE rates were already observed among children <5 years admitted to the MDH before to the vaccine introduction, a significant acceleration of the declining trends of AGE cases was observed when considering the entire study period, which may be attributable to various factors including improvement of healthcare, sanitation and adherence to the healthcare services by the population. The vaccine may have contributed in this acceleration, as there was approximately 40% reduction of expected cases which corroborate the GEMS findings that suggested that implementing the existing interventions (e.g. rotavirus vaccine) could prevent disease in approximately 35% of all diarrheal in infants [16]. In fact, a higher proportion of rotavirus-associated AGE prevented by the vaccine among infants was observed by 94% in our study. In addition, the decline of the rate of rotavirus associated AGE among children older than 2 years of age may suggest the beneficial or indirect effect of vaccination (e.g. herd immunity) as demonstrated in previous studies [24], [25], [26].

Despite the overall rotavirus AGE reduction that we have observed in successive years post-vaccine introduction, there was a slight increase of rotavirus frequency from 13% in 2018 to 18% in 2019, the third and fourth year after vaccine introduction. We do not have a plausible explanation for this finding, despite that early reports have documented rotaviruses to be more prevalent in HIV infected children compared uninfected ones (23.3%, 10/43 *vs.* 2.9%, 2/70; p < 0.0001) [27]. One of the limitation of our study is the lack of consistent data to rule out this hypothesis, despite that we previously documented 25% of children with MSD co-infected by HIV [12]. Besides, this increase also may suggest that infants children may be driving disease transmission across the study population, as stated by previous studies which observed the same trend [24].

We did find statistical significance on the longitudinal effect of the rotavirus vaccine against the prevalence of all-cause hospital admissions, AGE and LC-RV, although there was no statistical difference in malnutrition. The trends of malnutrition, may be related to the positive results of the multi-sectorial (health, education, social, agriculture, industry and commerce and public workers and housing sector) action plans implemented by Mozambican government which aimed to reduce the burden from 44% in 2008 to 30% in 2015 and 20% in 2020, combining activities through various sectors with activities implemented by Food and Nutrition Security Strategy (ESAN II) and the Action Plan for Food and Nutrition Security (PASAN II) [28]. And we do observed declines in the acute malnutrition among our study community, from 200 to 2010, with absolute number of admissions ranging from 400 cases observed in 2003 and reduction to 150 cases in 2010 [29].

Our findings of decline of all-cause hospitalizations and rotavirus-associated AGE are consistent with early observations from African countries within the African Rotavirus Surveillance Network (ARSN), which also documented declines in the proportion of hospitalizations due to rotavirus AGE [30]. The dropping on rotavirus detection rate documented in our study has also been reported in Mozambique in urban areas of Maputo, Beira, Quelimane and Nampula city, by de Deus *et al.*, from 38.3% before to 13.5% after vaccine introduction [4]. Even with socio-economic differences of our rural community with the urban areas.

We observed a possible delay of rotavirus seasonality before vaccine introduction similar to what has been documented by studies, in African Countries such as Kenya, also in the United States [31], [32], and reports from Mozambique [4]. We believe that COVID-19 pandemic may have impacted our study, when comparing the diarrheal cases reported in 2018 and 2019 before COVID-19 cases in Mozambique, although we may not attribute these figures only to COVID-19, as we were observing a slight decrease of diarrheal cases from 2018 to 2019.

We documented a significant reduction in rotavirus positivity and all-cause of diarrhea hospitalizations after vaccine introduction in a rural setting of southern Mozambique, suggesting the clear beneficial effect of vaccination, including its indirect effect (probable herd effect) in older children.

### CRediT authorship contribution statement

**Filomena Manjate:** Conceptualization, Methodology, Investigation, Project administration, Writing – original draft, Writing – review & editing. **Llorenç Quintó:** Conceptualization, Formal analysis, Writing – review & editing. **Percina Chirinda:** Conceptualization, Writing – review & editing. **Sozinho Acácio:** Conceptualization, Writing – review & editing. **Marcelino Garrine:** Conceptualization, Writing – review & editing. **Delfino Vubil:** Conceptualization, Writing – review & editing. **Tacilta Nhampossa:** Conceptualization, Writing – review & editing. **Eva D. João:** Conceptualization, Writing – review & editing. **Arsénio Nhacolo:** Conceptualization, Formal analysis, Writing – review & editing. **Anelsio Cossa:** Conceptualization, Writing – review & editing. **Sérgio Massora:** Conceptualization, Writing – review & editing. **Gizela Bambo:** Conceptualization, Writing – review & editing. **Quique Bassat:** Conceptualization, Writing – review & editing. **Karen Kotloff:** Conceptualization, Investigation, Project administration, Writing – review & editing. **Myron Levine:** Conceptualization, Writing – review & editing. **Pedro L. Alonso:** Conceptualization, Investigation, Project administration, Writing – review & editing. **Jacqueline E. Tate:** Conceptualization, Writing – review & editing. **Umesh Parashar:** Conceptualization, Writing – review & editing. **Jason M. Mwenda:** Conceptualization, Writing – review & editing. **Inácio Mandomando:** Conceptualization, Methodology, Investigation, Project administration, Writing – original draft, Writing – review & editing.

## Declaration of Competing Interest

The authors declare that they have no known competing financial interests or personal relationships that could have appeared to influence the work reported in this paper.
