# Supplementary material for: Impact of rotavirus vaccination on diarrheal hospitalizations in children younger than 5 years of age in a rural southern Mozambique
Source: Vaccine. 2022 Oct 19;40(44):6422–30. doi: 10.1016/j.vaccine.2022.09.050 (PMC9589241; doi:10.1016/j.vaccine.2022.09.050)
Supplement: Supplementary data 1 [file mmc1.docx]

**Supplementary data: Figures**

**
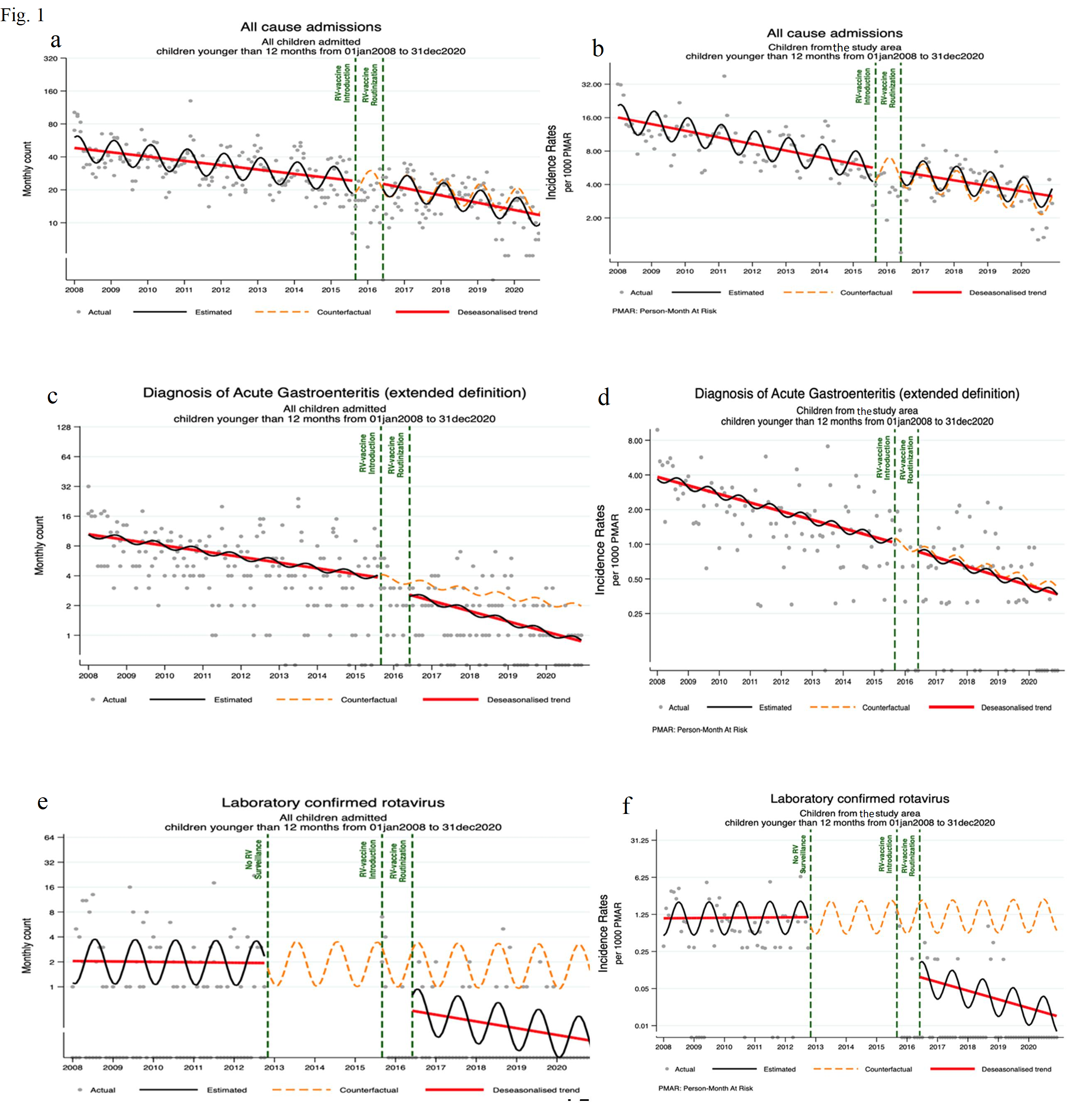
**

**Supplementary figure 1**. Trend of prevalence and incidence rates among children less than 12 months of age from January 01, 2008 – December 2020 Manhiça District, Mozambique (**a**) Trend over time of hospital admissions due to acute gastroenteritis (**b**) trend over time of the incidence rate of hospital admissions due to acute gastroenteritis (**c**) trend over time of diagnosis of acute gastroenteritis (**d**) trend over time of the incidence rate of diagnosis of acute gastroenteritis (**e**) trend over time of rotavirus confirmed cases (**f**) trend over time of the incidence rate of rotavirus confirmed cases.

**
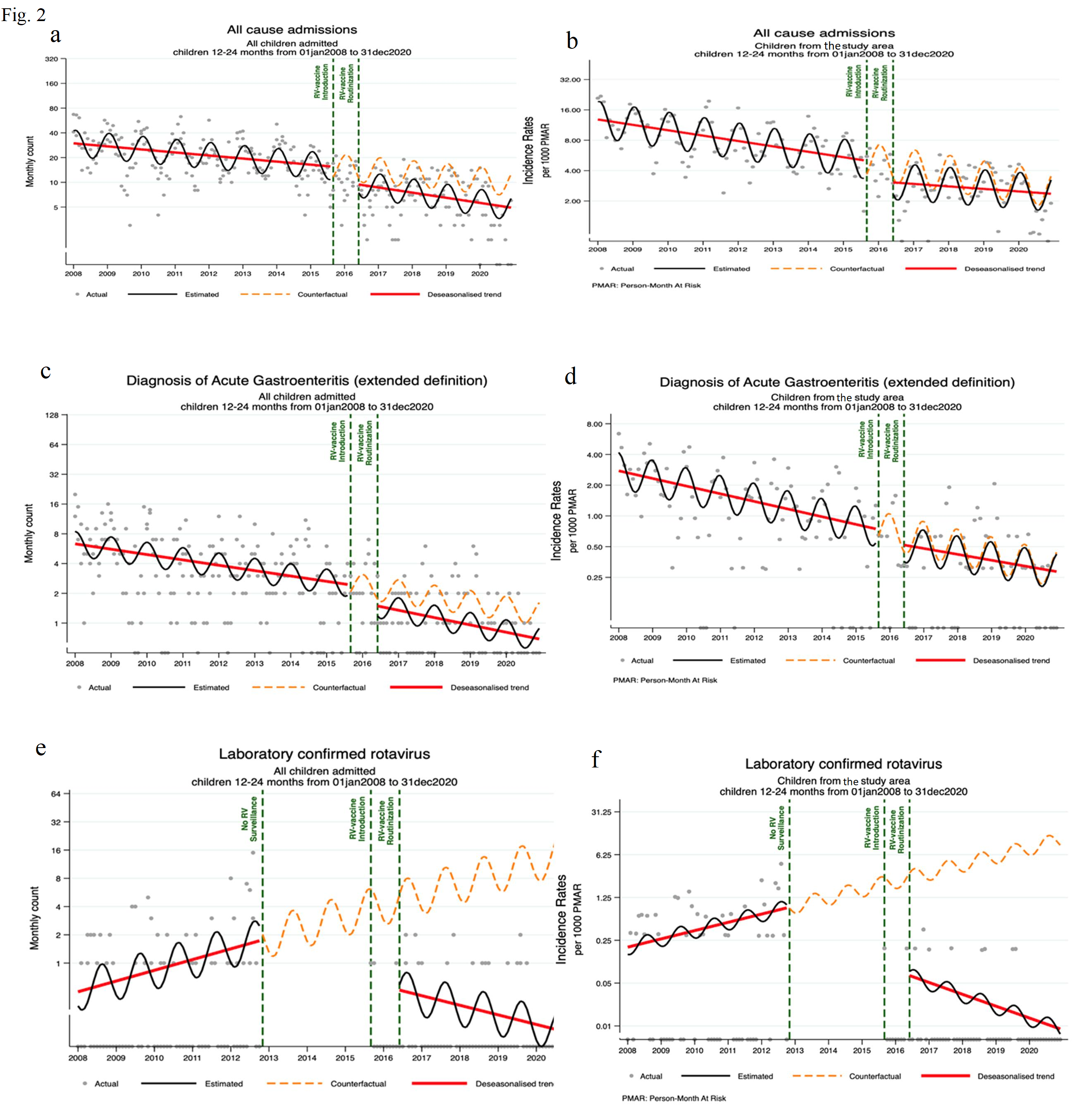
**

**Supplementary figure 2**. Trend of prevalence and incidence rates among children from 12-23 months of age from January 01, 2008 – December, Manhiça District, Manhiça Mozambique. (**a**) Trend over time of hospital admissions due to acute gastroenteritis (**b**) trend over time of the incidence rate of hospital admissions due to acute gastroenteritis (**c**) trend over time of diagnosis of acute gastroenteritis (**d**) trend over time of the incidence rate of diagnosis of acute gastroenteritis (**e**) trend over time of rotavirus confirmed cases (**f**) trend over time of the incidence rate of rotavirus confirmed cases.

**
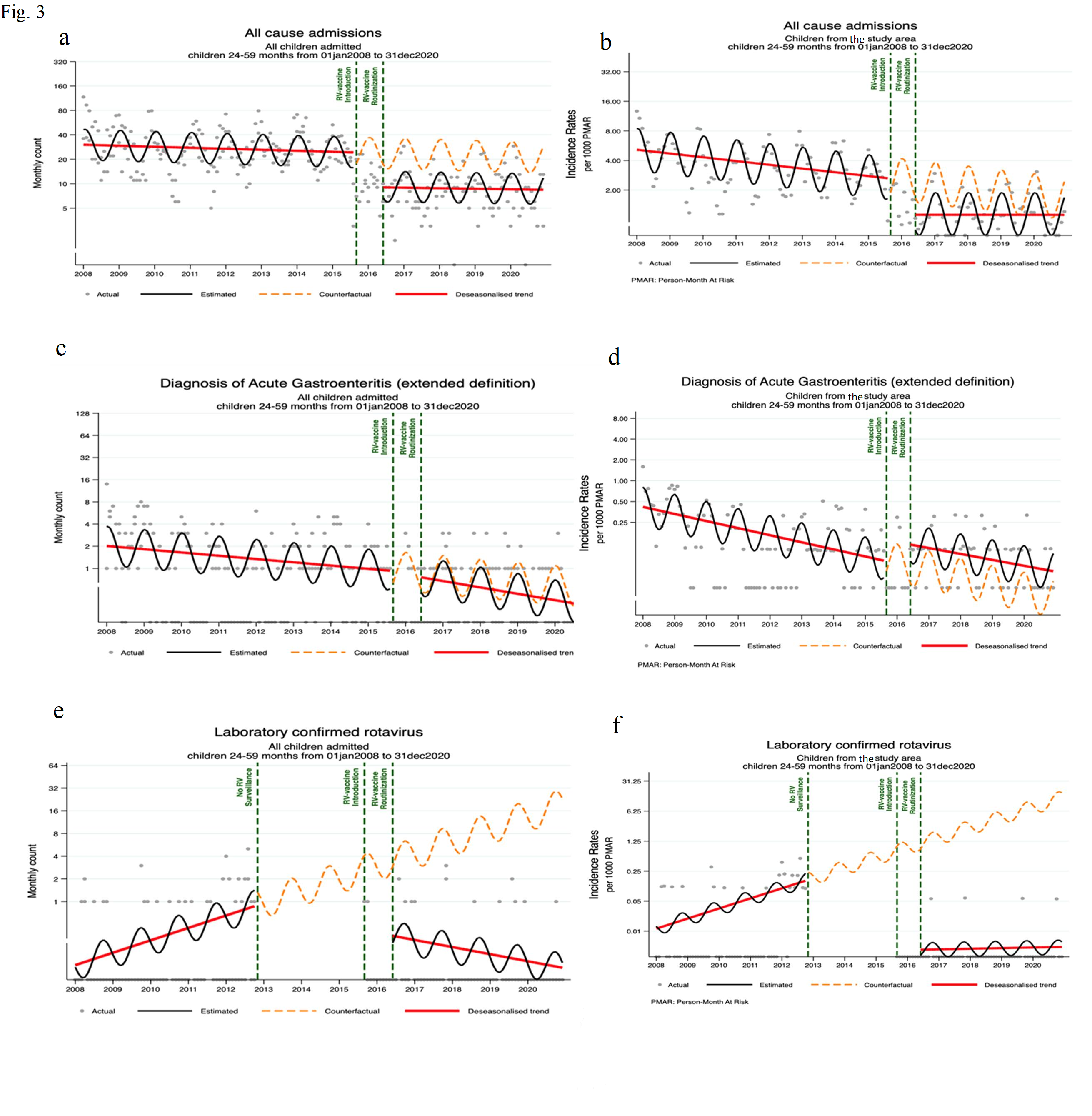
**

**Supplementary figure 3**. Trend of prevalence and incidence rates among children from 24-59 months of age from January 01, 2008 – December 2020, Manhiça District, Manhiça Mozambique. (**a**) Trend over time of hospital admissions due to acute gastroenteritis (**b**) trend over time of the incidence rate of hospital admissions due to acute gastroenteritis (**c**) trend over time of diagnosis of acute gastroenteritis (**d**) trend over time of the incidence rate of diagnosis of acute gastroenteritis (**e**) trend over time of rotavirus confirmed cases (**f**) trend over time of the incidence rate of rotavirus confirmed cases.

**
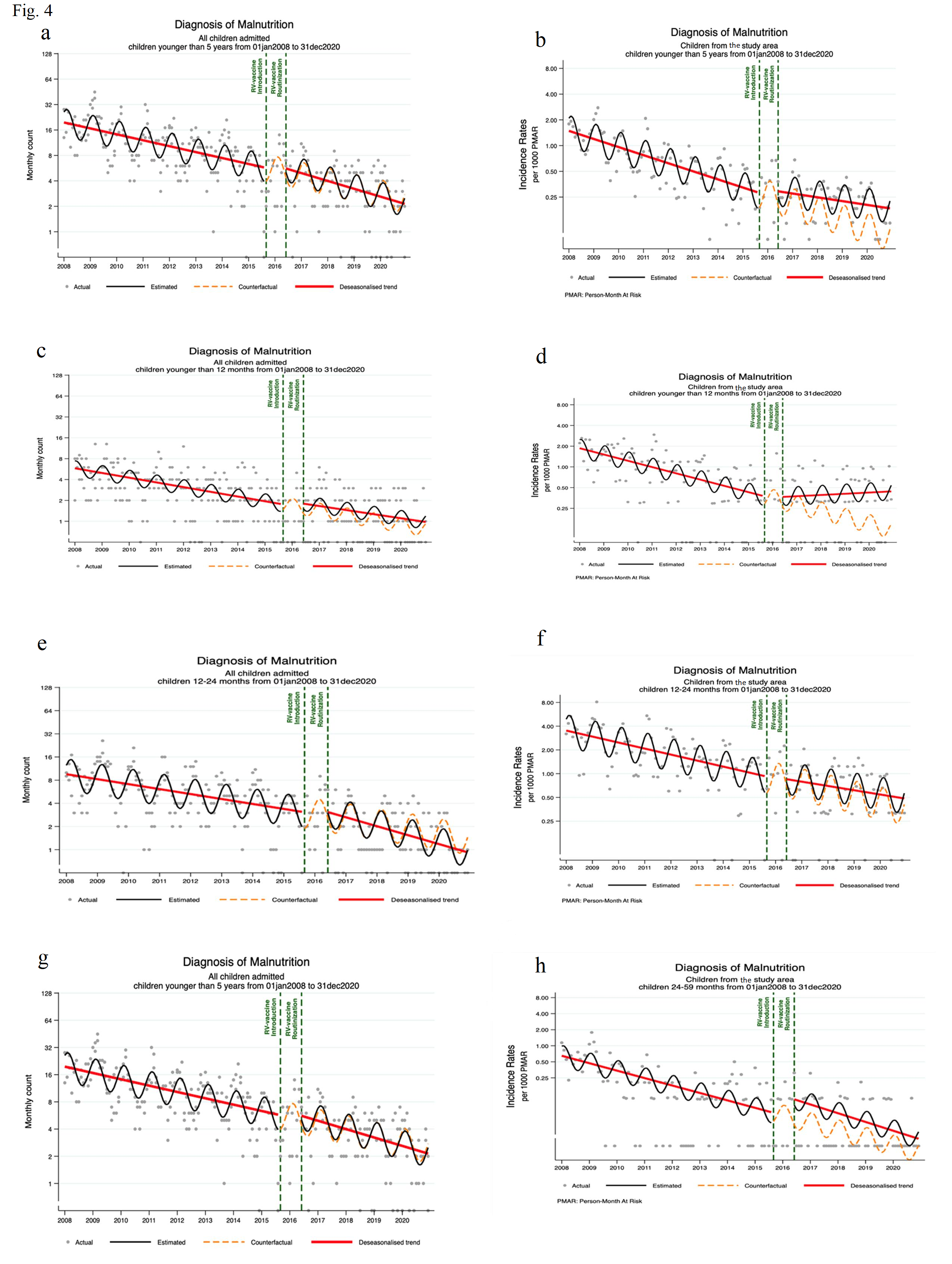
**

**Supplementary figure 4**. Trend of prevalence and incidences rates of malnutrition in children < 5 years of age and stratified by the age groups from January 01, 2008 – December 2020, Manhiça District, Manhiça Mozambique. (**a**) Trend over time of diagnosis of malnutrition among children < 5 years of age (**b**) trend over time of the incidence rate of diagnosis of malnutrition among children <5 years of age (**c**) trend over time diagnosis of malnutrition in children < 12 months of age (**d**) trend over time of the incidence rate of malnutrition among children < 12 months of age (**e**) trend over time diagnosis of malnutrition children from 12-23 months of age (**f**) trend over time o incidence rate of malnutrition among children from 12-23 months of age (**g**) trend over time diagnosis of malnutrition in children from 24-59 months of age (**h**) trend over time o incidence rate of malnutrition among children from 24-59 months of age

Fig. 5


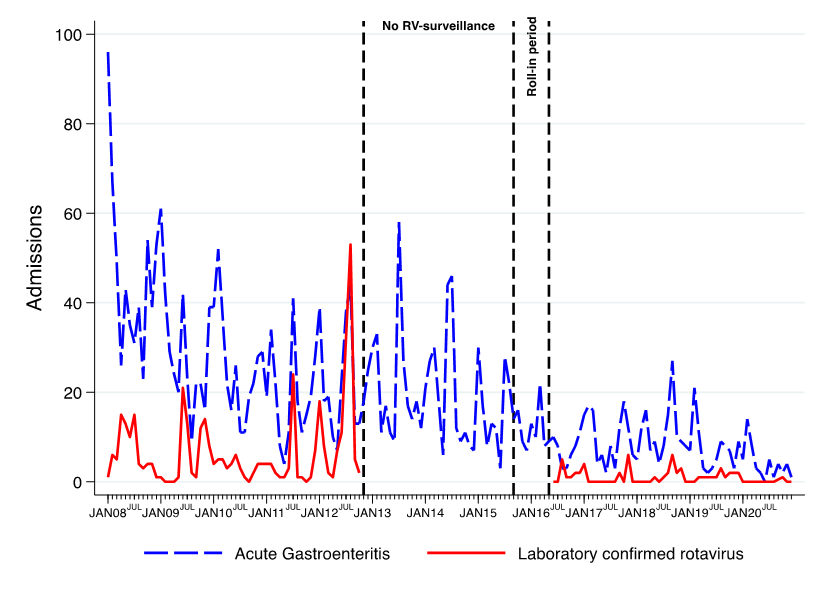


**Supplementary figure 5**. Trend over time of monthly acute gastroenteritis admissions, and laboratory confirmed rotavirus cases among children less than 5 years of age in Manhiça District, January 2008 – December 2020
