# Supplementary material for: Impact of rotavirus vaccination on diarrheal hospitalizations in children younger than 5 years of age in a rural southern Mozambique
Source: Vaccine. 2022 Oct 19;40(44):6422–30. doi: 10.1016/j.vaccine.2022.09.050 (PMC9589241; doi:10.1016/j.vaccine.2022.09.050)
Supplement: Supplementary data 2 [file mmc2.docx]

**Supplementary data: Tables**

**Supplementary table 1**. Estimated prevalence of malnutrition in all admitted children younger than 5 years of age stratified by age groups admitted to the Manhiça District Hospital, Manhiça Mozambique January 2008 – December 2020,

| Age category/Exposure | Admissions from all causes | Episodes of malnutrition | Rate estimates | |  | Model estimates | | p-value |
| --- | --- | --- | --- | --- | --- | --- | --- | --- |
|  |  |  | Prevalence (%) | 95% Conf. Interval |  | Odds Ratio | 95% Conf. Interval |  |
|  |  |  |  |  |  |  |  |  |
| **0-11 months** |  |  |  |  |  |  |  |  |
|  |  |  |  |  |  |  |  |  |
| Pre-vaccine introduction | 4614 | 477 | 10.3 | (9.5, 11.2) |  | 1 |  |  |
| Post-vaccine introduction | 2152 | 193 | 8.9 | (7.8, 10.2) |  | 0.9 | (0.7, 1.0) | 0.0792 |
| TOTAL | 6766 | 670 | 9.9 | (9.2, 10.6) |  | - |  |  |
|  |  |  |  |  |  |  |  |  |
| **12-23 months** |  |  |  |  |  |  |  |  |
| Pre-vaccine introduction | 2998 | 853 | 28.4 | (26.8, 30.1) |  | 1 |  |  |
| Post-vaccine introduction | 928 | 249 | 26.8 | (24.0, 29.8) |  | 0.9 | (0.8, 1.0) | 0.3371 |
| TOTAL | 3926 | 1102 | 28 | (26.6, 29.5) |  | - |  |  |
|  |  |  |  |  |  |  |  |  |
| **24-59 months** |  |  |  |  |  |  |  |  |
| Pre-vaccine introduction | 3464 | 309 | 8.9 | (7.9, 9.9) |  | 1 |  |  |
| Post-vaccine introduction | 1205 | 71 | 5.9 | (4.6, 7.3) |  | 0.6 | (0.5, 0.8) | 0.001 |
| TOTAL | 4669 | 380 | 8.1 | (7.3, 8.9) |  | - |  |  |
|  |  |  |  |  |  |  |  |  |
| **All age strata** |  |  |  |  |  |  |  |  |
| Pre-vaccine introduction | 11076 | 1639 | 14.8 | (14.1, 15.5) |  | 1 |  |  |
| Post-vaccine introduction | 4285 | 513 | 11.9 | (11.0, 13.0) |  | 0.8 | (0.7, 0.9) | <0.0001 |
| TOTAL | 15361 | 2152 | 14.0 | (13.4, 14.6) |  | - |  |  |

**Supplementary table 2.** Estimated incidence rates of malnutrition among children younger than 5 years of age stratified by age groups, admitted to the Manhiça District Hospital, Manhiça Mozambique January 2008 – December 2020,

| Age category/Exposure | Subjects | Episodes of malnutrition | Time At Risk (CYAR) | Rate estimates | |  | Model estimates | | p-value |
| --- | --- | --- | --- | --- | --- | --- | --- | --- | --- |
|  |  |  |  | Incidence Rate (per 1000 CYAR) | 95% Conf. Interval |  | Incidence Rate Ratio | 95% Conf. Interval |  |
|  |  |  |  |  |  |  |  |  |  |
| **0-11 months** |  |  |  |  |  |  |  |  |  |
|  |  |  |  |  |  |  |  |  |  |
| Pre-vaccine introduction | 21259 | 229 | 16033.58 | 14.2 | (12.6, 16.2) |  | 1 |  |  |
| Post-vaccine introduction | 32682 | 111 | 25143.38 | 4.4 | (3.7, 5.3) |  | 0.3 | (0.2, 0.4) | <0.0001 |
| TOTAL | 53941 | 340 | 41176.96 | 8.2 | (7.4, 9.1) |  | - |  |  |
|  |  |  |  |  |  |  |  |  |  |
| **12-23 months** |  |  |  |  |  |  |  |  |  |
| Pre-vaccine introduction | 20584 | 472 | 15624.2 | 30.2 | (27.6, 33.0) |  | 1 |  |  |
| Post-vaccine introduction | 33187 | 165 | 25382.11 | 6.5 | (5.6, 7.6) |  | 0.2 | (0.2, 0.3) | <0.0001 |
| TOTAL | 53771 | 637 | 41006.31 | 15.5 | (14.3, 16.8) |  | - |  |  |
|  |  |  |  |  |  |  |  |  |  |
| **24-59 months** |  |  |  |  |  |  |  |  |  |
| Pre-vaccine introduction | 27731 | 183 | 46078.37 | 3.9 | (3.4, 4.6) |  | 1 |  |  |
| Post-vaccine introduction | 49386 | 43 | 79834.3 | 0.5 | (0.4, 0.7) |  | 0.1 | (0.1, 0.2) | <0.0001 |
| TOTAL | 77117 | 226 | 125912.7 | 1.7 | (1.6, 2.0) |  | - |  |  |
|  |  |  |  |  |  |  |  |  |  |
| **All age strata** |  |  |  |  |  |  |  |  |  |
| Pre-vaccine introduction | 37350 | 884 | 77736.14 | 11.3 | (10.7, 12.1) |  | 1 |  |  |
| Post-vaccine introduction | 63372 | 319 | 130359.8 | 2.4 | (2.1, 2.7) |  | 0.2 | (0.2, 0.2) | <0.0001 |
| TOTAL | 96351 | 1203 | 208095.9 | 5.7 | (5.4, 6.1) |  | - |  |  |

**Supplementary table 3A**. Longitudinal effect of rotavirus vaccination on the number of hospitalizations, acute gastroenteritis, malnutrition and laboratory confirmed rotavirus among children <12 months of age adjusted for seasonality, admitted to the Manhiça District Hospital, Manhiça Mozambique January 2008 – December 2020

| Variable | Prevalence | | | Incidence | | |
| --- | --- | --- | --- | --- | --- | --- |
|  | Coef. | (95% Conf. Interval) | p-value | Coef. | (95% Conf. Interval) | p-value |
| **0-11 months** |  |  |  |  |  |  |
| **All cause admissions** |  |  |  |  |  |  |
| Baseline level (Intercept) | 3.88 | (3.70, 4.06) | < 0.0001 | -4.12 | (-4.24, -4.01) | < 0.0001 |
| Baseline monthly trend | -0.01 | (-0.01, 0) | 0.0196 | -0.01 | (-0.01, -0.01) | < 0.0001 |
| Level change after RV-vaccine introduction | 0.03 | (-0.14, 0.21) | 0.7076 | 0.05 | (-0.13, 0.23) | 0.5933 |
| Monthly trend change after RV-vaccine introduction | -0.01 | (-0.02, 0.01) | 0.4461 | 0 | (0.01) | 0.4684 |
|  |  |  |  |  |  |  |
| **Diagnosis of acute gastroenteritis** |  |  |  |  |  |  |
| Baseline level (Intercept) | 2.37 | (1.99, 2.74) | < 0.0001 | -5.55 | (-5.71, -5.38) | < 0.0001 |
| Baseline monthly trend | -0.01 | (-0.02, -0.005) | 0.0003 | -0.01 | (-0.02, -0.01) | < 0.0001 |
| Level change after RV-vaccine introduction | -0.30 | (-0.89, 0.30) | 0.3282 | -0.06 | (-0.46, 0.35) | 0.7851 |
| Monthly trend change after RV-vaccine introduction | -0.01 | (-0.02, 0) | 0.0337 | 0 | (-0.01, 0.01) | 0.7700 |
|  |  |  |  |  |  |  |
| **Diagnosis of malnutrition** |  |  |  |  |  |  |
| Baseline level (Intercept) | 1.77 | (1.54, 2.01) | < 0.0001 | -6.27 | (-6.50, -6.03) | < 0.0001 |
| Baseline monthly trend | -0.01 | (-0.02, 0) | 0,0105 | -0.02 | (-0.02, -0.01) | < 0.0001 |
| Level change after RV-vaccine introduction | 0.15 | (-0.08, 0.39) | 0.2029 | 0.14 | (-0.43, 0.70) | 0.6380 |
| Monthly trend change after RV-vaccine introduction | 0 | (-0.03, 0.03) | 0.9104 | 0,02 | (0.01, 0.4) | 0.0078 |
|  |  |  |  |  |  |  |
| **Laboratory confirmed rotavirus** |  |  |  |  |  |  |
| Baseline level (Intercept) | 0.71 | (-0.97, 2.39) | 0.4063 | -6.88 | (-7.28, -6.47) | < 0.0001 |
| Baseline monthly trend | 0 | (-0.00, -0.000) | 0.0180 | 0 | (-0.01, 0.01) | 0.8794 |
| Level change after RV-vaccine introduction | -1.26 | (-2.12, -0.39) | 0.0043 | -2.61 | (-3.61, -1.61) | < 0.0001 |
| Monthly trend change after RV-vaccine introduction | -0.02 | (-0.02, -0.01) | 0.0003 | -0.03 | (-0.06, 0) | 0.0289 |
|  |  |  |  |  |  |  |

**Supplementary table 3B**. Longitudinal effect of rotavirus vaccination on the number of hospitalizations, acute gastroenteritis, malnutrition and laboratory confirmed rotavirus among children from 12-23 months of age adjusted for seasonality, admitted to the Manhiça District Hospital, Manhiça Mozambique January 2008 – December 2020

| Variable | Prevalence | | | Incidence | | |
| --- | --- | --- | --- | --- | --- | --- |
|  | Coef. | (95% Conf. Interval) | p-value | Coef. | (95% Conf. Interval) | p-value |
| **12-23 months** |  |  |  |  |  |  |
| **All cause admissions** |  |  |  |  |  |  |
| Baseline level (Intercept) | 3.40 | (2.83, 3.98) | < 0.0001 | -4.35 | (-4.48, -4.21) | < 0.0001 |
| Baseline monthly trend | -0.01 | (-0.01, 0) | 0.0015 | -0.01 | (-0.01, -0.01) | < 0.0001 |
| Level change after RV-vaccine introduction | -0.42 | (-0.50, -0.33) | < 0.0001 | -0.39 | (-0.57, -0.20) | < 0.0001 |
| Monthly trend change after RV-vaccine introduction | -0.01 | (-0.02, 0.01) | 0.5851 | 0.01 | (0, 0.01) | 0.0730 |
|  |  |  |  |  |  |  |
| **Diagnosis of acute gastroenteritis** |  |  |  |  |  |  |
| Baseline level (Intercept) | 1.86 | (1.26, 2.46) | < 0.0001 | -5.87 | (-6.03, -5.71) | < 0.0001 |
| Baseline monthly trend | -0.01 | (-0.02, -0.01) | < 0.0001 | -0.01 | (-0.02, -0.01) | < 0.0001 |
| Level change after RV-vaccine introduction | -0.39 | (-0.61, -0.17) | 0.0005 | -0.22 | (-0.69, 0.25) | 0.3592 |
| Monthly trend change after RV-vaccine introduction | 0 | (-0.01, 0) | 0.2728 | 0 | (-0.01, 0.02) | 0.5699 |
|  |  |  |  |  |  |  |
| **Diagnosis of malnutrition** |  |  |  |  |  |  |
| Baseline level (Intercept) | 2.26 | (1.95, 2.57) | < 0.0001 | -5.64 | (-5.79, -5.50) | < 0.0001 |
| Baseline monthly trend | -0.01 | (-0.02, -0.01) | < 0.0001 | -0.02 | (-0.02, -0.01) | < 0.0001 |
| Level change after RV-vaccine introduction | 0.16 | (-0.09, 0.42) | 0.2110 | 0.09 | (-0.34, 0.53) | 0.6692 |
| Monthly trend change after RV-vaccine introduction | -0.01 | (-0.04, 0.02) | 0.5342 | 0 | (-0.01, 0.02) | 0.5453 |
|  |  |  |  |  |  |  |
| **Laboratory confirmed rotavirus** |  |  |  |  |  |  |
| Baseline level (Intercept) | -0.71 | (-1.97, 0.56) | 0.2726 | -8.58 | (-9.18, -7.99) | < 0.0001 |
| Baseline monthly trend | 0.02 | (0.01, 0.03) | < 0.0001 | 0.03 | (0.01, 0.041) | 0.0007 |
| Level change after RV-vaccine introduction | -2.17 | (-3.39, -0.96) | 0.0005 | -3.63 | (-5.06, -2.20) | < 0.0001 |
| Monthly trend change after RV-vaccine introduction | -0.04 | (-0.04, -0.04) | < 0.0001 | -0.06 | (-0.11, -0.011) | 0.0060 |
|  |  |  |  |  |  |  |

**Supplementary table 3C**. Longitudinal effect of rotavirus vaccination on the number of hospitalizations, acute gastroenteritis, malnutrition and laboratory confirmed rotavirus among children from 24-59 months of age adjusted for seasonality, admitted to the Manhiça District Hospital, Manhiça Mozambique January 2008 – December 2020

| Variable | Prevalence | | | Incidence | | |
| --- | --- | --- | --- | --- | --- | --- |
|  | Coef. | (95% Conf. Interval) | p-value | Coef. | (95% Conf. Interval) | p-value |
| **24-59 months** |  |  |  |  |  |  |
| **All cause admissions** |  |  |  |  |  |  |
| Baseline level (Intercept) | 3.41 | (2.65, 4.17) | < 0.0001 | -5.26 | (-5.45, -5.08) | < 0.0001 |
| Baseline monthly trend | 0 | (-0.01, 0) | 0.3769 | -0.01 | (-0.01, -0.01) | < 0.0001 |
| Level change after RV-vaccine introduction | -0.95 | (-1.19, -0.70) | < 0.0001 | -0.77 | (-0.99, -0.55) | < 0.0001 |
| Monthly trend change after RV-vaccine introduction | 0 | (-0.01, 0.01) | 0.8038 | 0.01 | (0, 0.01) | 0,0187 |
|  |  |  |  |  |  |  |
| **Diagnosis of acute gastroenteritis** |  |  |  |  |  |  |
| Baseline level (Intercept) | 0.71 | (-0.38, 1.80) | 0.2005 | -7.76 | (-8.04, -7.45) | < 0.0001 |
| Baseline monthly trend | -0.01 | (-0.02, 0.1) | 0.2545 | -0.02 | (-0.03, -0.01) | **< 0.0001** |
| Level change after RV-vaccine introduction | -0.09 | (-1.55, 1.36) | 0.9023 | 0.74 | (0.12, 1.32) | 0,0100 |
| Monthly trend change after RV-vaccine introduction | -0.1 | (-0.02, 0) | 0.2027 | 0 | (-0.01, 0.02) | 0,7173 |
|  |  |  |  |  |  |  |
| **Diagnosis of malnutrition** |  |  |  |  |  |  |
| Baseline level (Intercept) | 1.41 | (0.73, 2.09) | < 0.0001 | -7.32 | (-7.5, -7.9) | < 0.0001 |
| Baseline monthly trend | -0.02 | (-0.03, 0) | 0.0086 | -0.03 | (-0.03, -0.02) | < 0.0001 |
| Level change after RV-vaccine introduction | 0.02 | (-1.08, 1.12) | 0.9759 | 0.85 | (-0.35, 2.06) | 0.1635 |
| Monthly trend change after RV-vaccine introduction | 0 | (-0.01, 0.01) | 0.9089 | -0.01 | (-0.04, 0.03) | 0.7868 |
|  |  |  |  |  |  |  |
| **Laboratory confirmed rotavirus** |  |  |  |  |  |  |
| Baseline level (Intercept) | -1.97 | (-2.08, -1.86) | < 0.0001 | -11.41 | (-12.62, -10.20) | < 0.0001 |
| Baseline monthly trend | 0.03 | (0, 0.06) | 0.039 | 0.05 | (0.02, 0.07) | 0.0018 |
| Level change after RV-vaccine introduction | -2.28 | (-4.94, 0.37) | 0.092 | -5.67 | (-8.64, -2.70) | 0.0002 |
| Monthly trend change after RV-vaccine introduction | -0.05 | (-0.06, -0.04) | < 0.0001 | -0.04 | (-0.12, 0.03) | 0.2618 |
|  |  |  |  |  |  |  |

**Supplementary table 4. ICD-10 code and definitions**

| ICD-10 Code | Definition |
| --- | --- |
| A02.0 | *Salmonella enteritis* |
| A02.1 | Salmonella sepsis |
| A02.9 | *Salmonella* infection, unspecified |
| A03.3 | Shigellosis due to *Shigella sonnei* |
| A03.9 | Shigellosis, unspecified |
| A04.4 | Other intestinal *Escherichia coli* infections |
| A04.5 | *Campylobacter enteritis* |
| A04.7 | Enterocolitis due to *Clostridium difficile* |
| A04.8 | Other specified bacterial intestinal infections |
| A04.9 | Bacterial intestinal infection, unspecified |
| A05.1 | Botulism |
| A07.2 | Cryptosporidiosis |
| A08.0 | Rotaviral enteritis |
| A08.1 | Acute gastroenteropathy due to Norwalk agent |
| A08.2 | Adenoviral enteritis |
| A08.3 | Other viral enteritis |
| A08.4 | Viral intestinal infection, unspecified Includes: viral enteritis not otherwise specified (NOS), viral gastroenteritis NOS, and viral gastroenteropathy NOS) |
|  |  |
| A08.5 | Other specified intestinal infections |
| A09 | Other gastroenteritis and colitis of infectious and unspecified origin Excludes: due to bacterial, protozoal, viral and other specified infectious agents (A00-A08) and noninfective (see noninfectious) diarrhoea (K52.9) |
| A09.0 | Other and unspecified gastroenteritis and colitis of infectious origin |
| A09.9 | Gastroenteritis and colitis of unspecified origin |
| K52.0 | Gastroenteritis and colitis due to radiation |
| K52.1 | Toxic gastroenteritis and colitis |
| K52.2 | Allergic and dietetic gastroenteritis and colitis |
| K52.8 | Other specified noninfective gastroenteritis and colitis |
| K52.9 | Noninfective gastroenteritis and colitis, unspecified Includes: diarrhea, enteritis, ileitis, jejunitis, and sigmoiditis if specified as noninfectious Excludes: colitis, diarrhoea, enteritis, gastroenteritis if coded as infectious (A09.0) or of unspecified origin (A09.9); functional diarrhoea (K59.1); neonatal diarrhoea (noninfective) (P78.3); psychogenic diarrhoea (F45.3) |
| E40 | Kwashiorkor |
| E41 | Nutritional marasmus |
| E42 | Marasmic kwashiorkor |
| E43 | Unspecified severe protein-calorie malnutrition |
| E44 | Protein-calorie malnutrition of moderate and mild degree |
| E45 | Retarded development following protein-calorie malnutrition |
| E46 | Unspecified protein-calorie malnutrition |

**Adapted from Wilson *et al*., 2015**
